# Supplementary material for: cONcat: Computational reconstruction of concatenated fragments from long Oxford Nanopore reads
Source: PLoS One. 2025 Jul 24;20(7):e0321246. doi: 10.1371/journal.pone.0321246 (PMC12289010; doi:10.1371/journal.pone.0321246)
Supplement: S1 File — (PDF) [file pone.0321246.s001.pdf]

Supplementary Material

1. DNA Library Preparation

We developed a diverse library of DNA fragments with varying sizes. To assemble the library, the fragments were annealed under a controlled temperature ramp, gradually cooling from 95°C to 4°C over a duration of 30 minutes. This process facilitated the precise hybridization of complementary strands.

The annealed fragments were subsequently ligated using T4 DNA ligase (New England Biolabs) at a 10h incubation at 16°C followed by an overnight incubation at 4°C, ensuring efficient covalent linkage between DNA fragments. This ligation step resulted in the creation of a heterogeneous library of double-stranded DNA (dsDNA).

The ligated DNA library was subjected to PCR amplification using primers complementary to the non-phosphorylated start and end sequences incorporated during the ligation reaction. These non-phosphorylated sequences functioned as terminal caps, preventing continuous extension of the ligated fragments and ensuring controlled amplification. A gradient PCR was performed to determine the optimal annealing temperature, with reactions conducted at 50°C, 53.6°C, 55°C, and 58°C for 15 cycles. Amplification was observed across all tested temperatures, and 58°C was selected for the final amplification to minimize non-specific primer binding. The PCR reaction setup is detailed in Table 1.

Component	Volume (µl)
2x DreamTaq Master Mix	100
Fwd primer-Start (10 µM)	10
Bwd primer-End (10 µM)	10
Ligated sample	2
H2O	78
Total Vol (ul)	200

Table 1: PCR Reaction Setup

Following amplification, the PCR products were purified using AMPure XP beads to remove unreacted components or smaller by-products ensuring high-purity amplicons for downstream nanopore sequencing. Sequencing was performed following the standardized protocol provided by the Nanopore Community, using the Flonglebranch of the Ligation Sequencing Amplicons V14 (SQK-LSK114) workflow.

Nanopore Sequencing Workflow

End-Preparation of DNA

For the end-prep reaction, 100 fmol of purified, ligated DNA was required. The DNA concentration was precisely measured using a Qubit fluorometer, assuming an average amplicon length of 0.5 kbp. Based on

this estimation, 32.5 ng of DNA was used to achieve the required input amount. The sample was diluted in nuclease-free water to a final volume of 24.5 µL before proceeding with the reaction setup, which was performed according to the recommended protocol (Table 1).

Reagent	Volume (µL)
DCS	0.5
DNA	24.5
Ultra II End-Prep Reaction Buffer	3.5
Ultra II End-Prep Enzyme Mix	1.5
Total	30

Table 2: End-Prep Reaction Setup

The reaction was incubated in a thermocycler at 20°C for 5 minutes, followed by 65°C for 5 minutes to complete the end-prep process. Upon completion, the sample was purified using AMPure XP beads and transferred to a clean 1.5 mL Eppendorf DNA LoBind tube for downstream processing.

AMPure Purification Protocol

1. **Bead Binding:** Add 30 µL of resuspended AMPure XP beads (AXP) to the end-prep reaction and mix gently by flicking the tube.
2. **Incubation:** Place the tube on a Hula mixer at room temperature for 5 minutes to allow DNA binding.
3. **Washing Steps:**
   - Prepare 500 µL of fresh 80% ethanol in nuclease-free water.
   - Spin down the sample and pellet the beads on a magnetic rack until the supernatant is clear.
   - Keeping the tube on the magnet, carefully remove the supernatant.
   - Wash the beads twice with 200 µL of 80% ethanol, ensuring the pellet remains undisturbed.
   - Remove any residual ethanol and allow the pellet to air-dry for ~30 seconds (avoiding over-drying to prevent cracking).
4. **Elution:**
   - Remove the tube from the magnetic rack and resuspend the pellet in 30 µL of nuclease-free water.
   - Incubate at room temperature for 2 minutes and return the tube to the magnetic rack.
   - Allow at least 1 minute for bead separation until the eluate is clear.
   - Carefully transfer 30 µL of eluate to a fresh 1.5 mL LoBind tube.

Adaptor Ligation and Purification

The entire purified DNA sample was used for the adaptor ligation step, following the recommended protocol (Table 2).

Reagent	Volume (µL)
DNA sample from the previous step	30
Ligation Buffer (LNB)	12.5
NEB Next Quick T4 DNA Ligase	5
Ligation Adapter (LA)	2.5

Total	50

Table 3: Adaptor Ligation Reaction Setup

1. **Adaptor Ligation Reaction:** The reaction was thoroughly mixed by gentle pipetting, briefly spun down, and incubated at room temperature for 10 minutes to facilitate ligation.
2. **AMPure XP Bead Purification:**
   - o The AMPure XP Beads were resuspended by vortexing.
   - o 20 µL of the resuspended AXP beads were added to the ligation reaction and mixed by flicking the tube.
   - o The mixture was incubated on a Hula mixer at room temperature for 5 minutes to allow DNA binding.
   - o The tube was spun down and placed on a magnetic rack until the beads formed a pellet. Once the supernatant was clear, it was carefully removed and discarded.
1. **Washing Steps:**
   - o 125 µL of Short Fragment Buffer (SFB) was added to the pellet, the tube was flicked to resuspend and then returned to the magnetic rack.
   - o Once the beads had pelleted, the supernatant was removed and discarded.
   - o The washing step was repeated to ensure complete removal of excess adaptors.
2. **Elution of DNA Library:**
   - o The tube was spun down and placed back on the magnet, with any residual supernatant removed.
   - o The pellet was allowed to air-dry for ~30 seconds, avoiding over-drying.
   - o The tube was removed from the magnetic rack, and the pellet was resuspended in 7 µL of Elution Buffer (EB).
   - o The mixture was incubated at room temperature for 10 minutes. For high molecular weight DNA, incubation at 37°C improved recovery.
   - o The tube was placed on the magnet until the eluate was clear and colorless (at least 1 minute).
   - o 7 µL of the purified DNA library was transferred to a clean 1.5 mL Eppendorf DNA LoBind tube, and the bead pellet was discarded.

3. **Final Quantification:**
   - The concentration of DNA sample was quantified before sequencing using 1ul of eluted DNA sample with Qubit fluorometer.

Final Preparation for Sequencing

Following purification, the sample was ready for sequencing. Before initiating the run, a flow cell quality check was performed to ensure optimal sequencing conditions. The sequencing reaction was set up as per ***Table 3***, and the Flow Cell ID was recorded. The MinION software was used to conduct a Flow Cell Check, where the number of active nanopores was assessed. The sample and pipettes were prepared for loading, ensuring proper handling to avoid contamination or air bubbles.

Reagent	Volume (µL)
Sequencing Buffer (SB)	15
Library Beads (LIB) (mixed immediately before use)	10
DNA Library	5

Total	30

Table 4: Final Library Preparation Setup

To flush the flow cell, 120 µL of flush buffer was loaded into the port. Before dispensing, the pipette plunger was unscrewed to remove any trapped air and expose a small drop of the sample. The pipette was held straight down into the exposed hole, ensuring a stable and secure fit before carefully dispensing the flush buffer by further unscrewing the plunger. Following the flush, 30 µL of the prepared DNA library was loaded using the same technique. Once the sample was successfully loaded, the sequencing run was initiated using MinION software, with the following settings applied like 20bp read length, POD5 detector and superaccurate basecalling. These settings ensured high-fidelity sequencing data, optimizing accuracy for downstream analysis.

2. Error per base values used for the first 100 bases of SIM_NU

To simulate a data set having non uniform error rates comparable to the error rates observed in actual ONT sequenced reads, we calculated the average error rate per base for the first 100 nucleotides of our experimental dataset. The average error rates per base for this range are as follows:
[0.51544048, 0.4633226, 0.41962698, 0.36491346, 0.37311439, 0.38849357, 0.41621851, 0.41683087, 0.41575156, 0.40372351, 0.3887138, 0.36476175, 0.33501046, 0.30455581, 0.27064415, 0.23457386, 0.20131726, 0.17431225, 0.15349413, 0.13793746, 0.12560718, 0.11617571, 0.10932199, 0.10399, 0.09931417, 0.09415376, 0.08774131, 0.07982219, 0.07201981, 0.06512025, 0.05915216, 0.05341532, 0.04848053, 0.0442655, 0.04047693, 0.03681451, 0.0334961, 0.03060112, 0.02820919, 0.02613725, 0.02442628, 0.02307134, 0.02214342, 0.02153789, 0.02130499, 0.0213524, 0.02154084, 0.02191075, 0.0223825, 0.02296152, 0.02362494, 0.02419089, 0.02464837, 0.02488377, 0.02482372, 0.02460933, 0.02430405, 0.02389571, 0.0234503, 0.02297452, 0.02250101, 0.02202724, 0.02161443, 0.02129141, 0.02116458, 0.02108961, 0.02120504, 0.02142005, 0.02169635, 0.02199924, 0.02225144, 0.02252378, 0.02270676, 0.02284495, 0.02293972, 0.022927, 0.02272802, 0.02245576, 0.02213147, 0.02186582, 0.02155404, 0.0212303, 0.02098836, 0.02080077, 0.02066826, 0.02060962, 0.02061027, 0.02063234, 0.02074351, 0.02086724, 0.02098664, 0.02112046, 0.02119616, 0.02121688, 0.02117194, 0.0211246, 0.02104943, 0.02096742, 0.02089215, 0.02085319]

3. Testing cONcat with simulated fragments with a fixed number of substitutions

To investigate the effect of fragment orthogonality on the performance of the cONcat algorithm, we simulated sets of fragments with different numbers of substitutions added per fragment *(n=1,2,4,6)*. Specifically, we simulated sets of 20 fragments, with each fragment of length 20nt, by inserting n mutations in each fragment. Four experiments using n=1, 2, 4, and 6. Following this, the expected range of edit distances between any pair of fragments is in *[1,2n],* as we do not accept exactly matching

fragments for this experiment. The resulting edit distance matrices are shown in (Suppl. Fig. 5, 8, 11, 14). We then simulated datasets of reads from each set of simulated fragments in the same manner as performed for SIM0-SIM30.

Additional figures

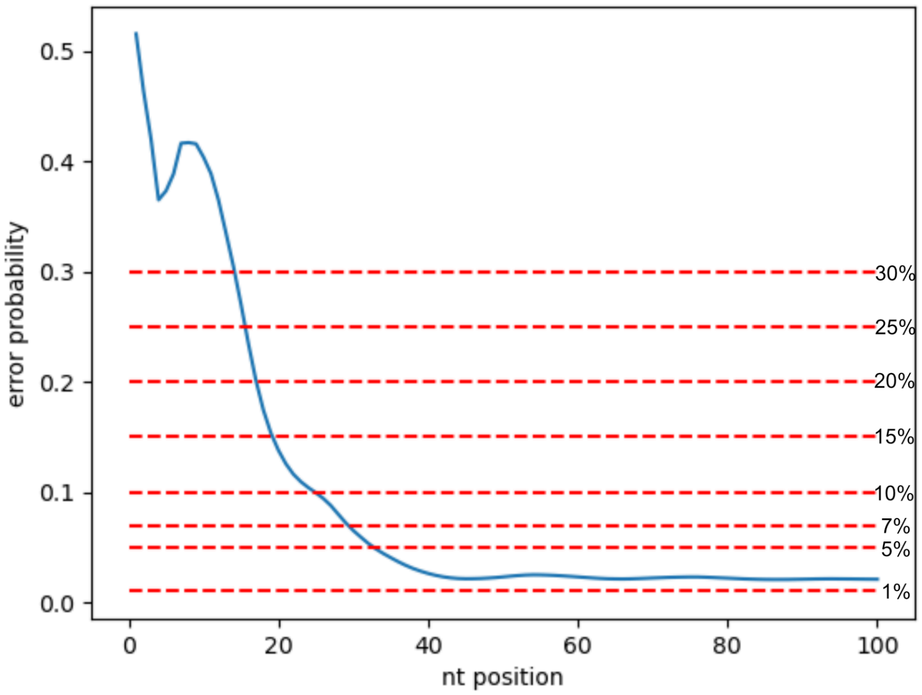

Supplementary figure 1. Error profile of experimental data and simulated constant error rate. The extracted error rate is plotted as a solid blue line. The simulated error rates are plotted as dashed red horizontal lines.

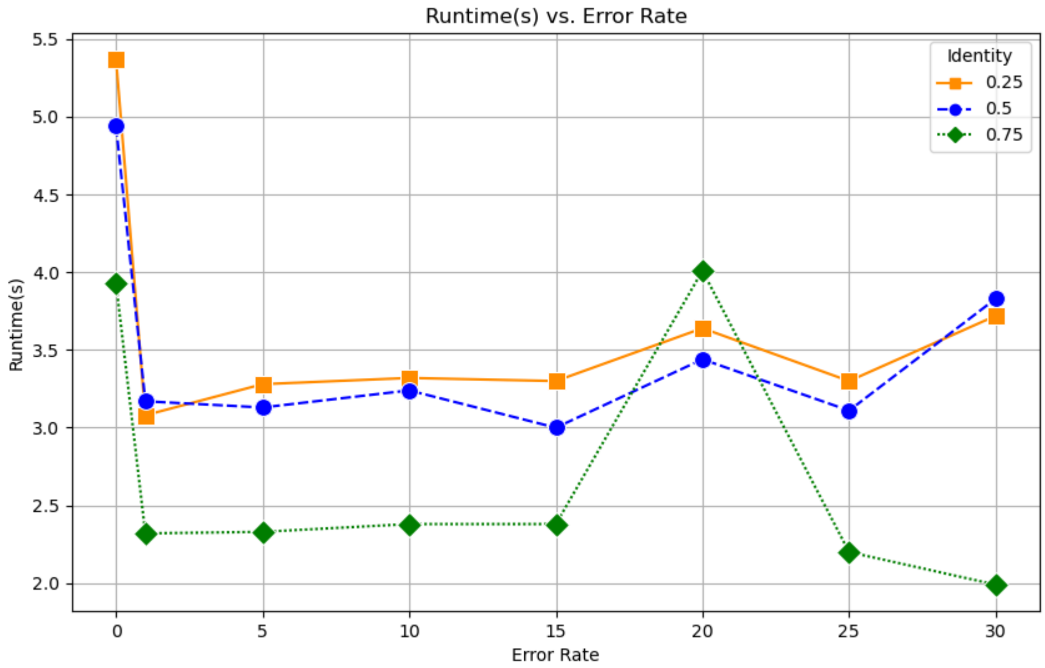

Supplementary figure 2. Runtime in seconds of the cONcat algorithm for the SIM_0-SIM_30 datasets. Overall the runtime of cONcat is low due to the algorithm efficiently working on the sequencing data. A trend of runtime increase can be observed with lower identity settings with the runtime of SIM_20 for 0.75 exceeding the lower settings for the same error rate. This might be due to the computer having different workloads during the run of this dataset.

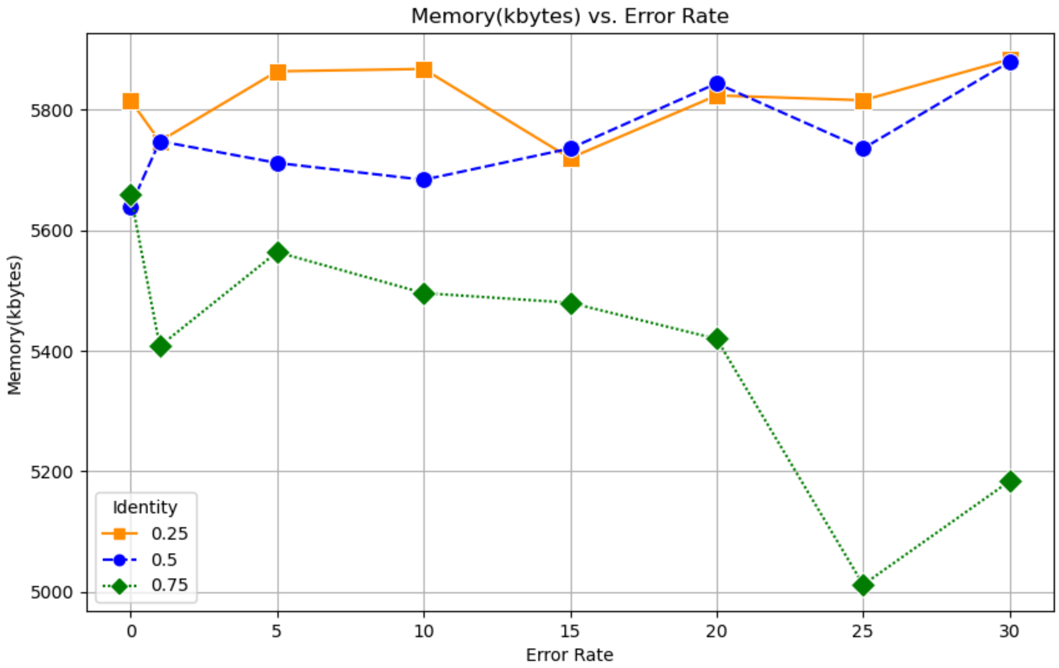

Supplementary figure 3. Memory usage in kilobytes for the SIM_0-SIM_30 datasets of the cONcat algorithm. Overall the memory usage of cONcat is low due to the algorithm not having to store much data. The memory usage, however, shows a trend of slightly increasing with lower identity settings due to a higher amount of fragment hits being considered.

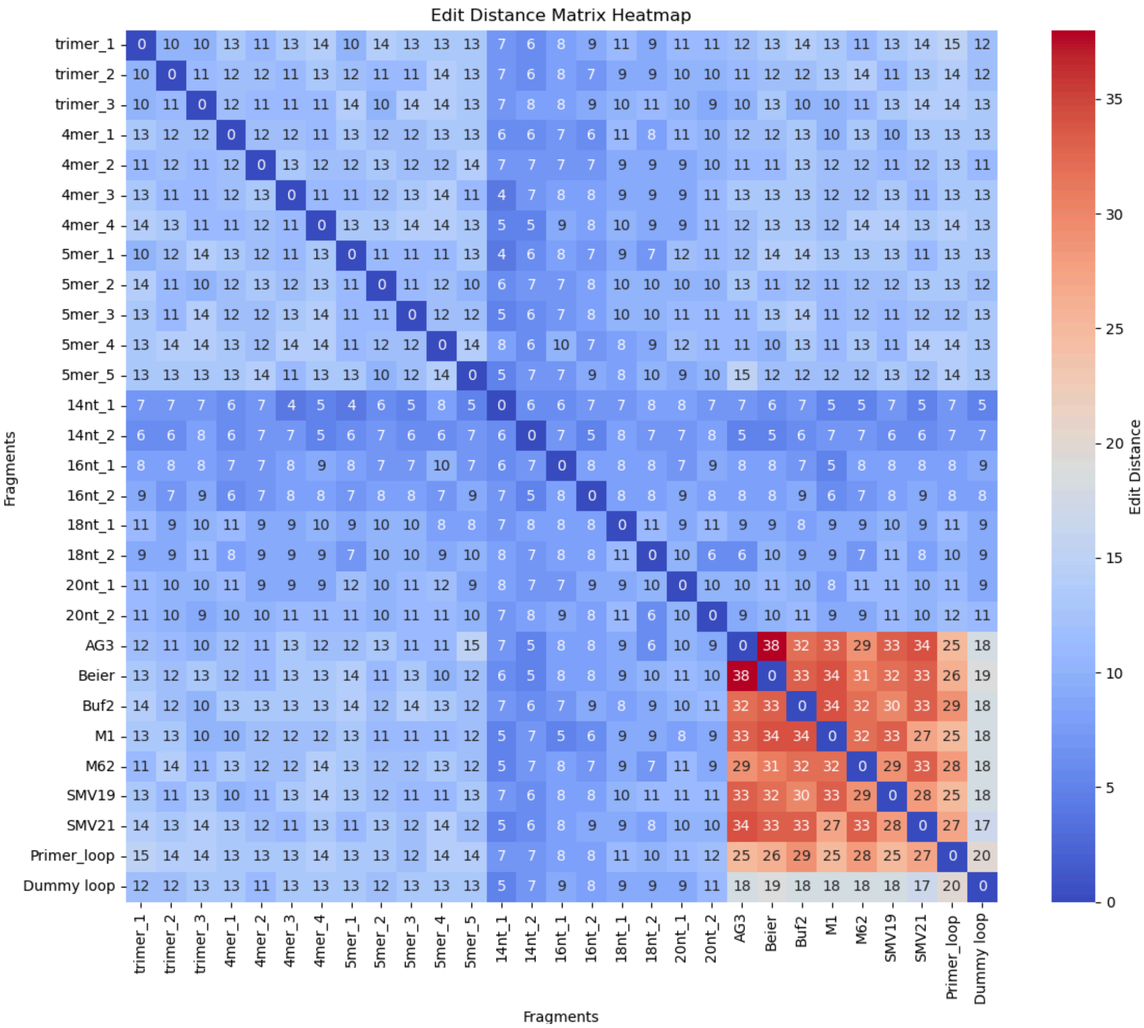

Supplementary Figure 4. Similarity matrix based on edit distance differences for the fragments used in the SIM_NU and the SIM0 to SIM30 experiments. We performed the edit distance calculations using the infix edit distance model (i.e., local alignment) to accommodate the different lengths of the fragments.

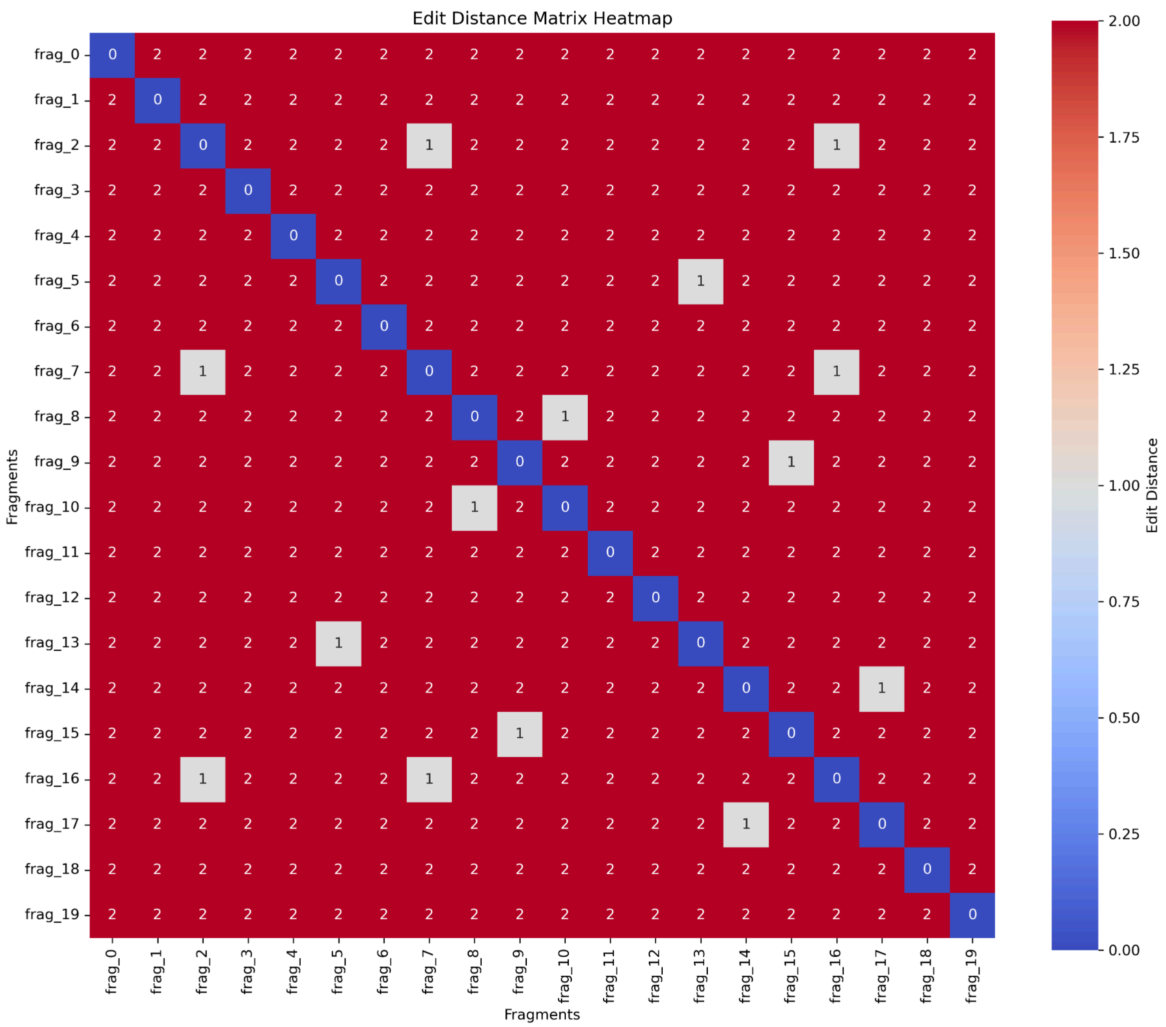

Supplementary figure 5. Edit distances for n = 1. While most pairs of fragments have edit distance 2, some fragments share a substitution and, therefore, only have an edit distance of 1.

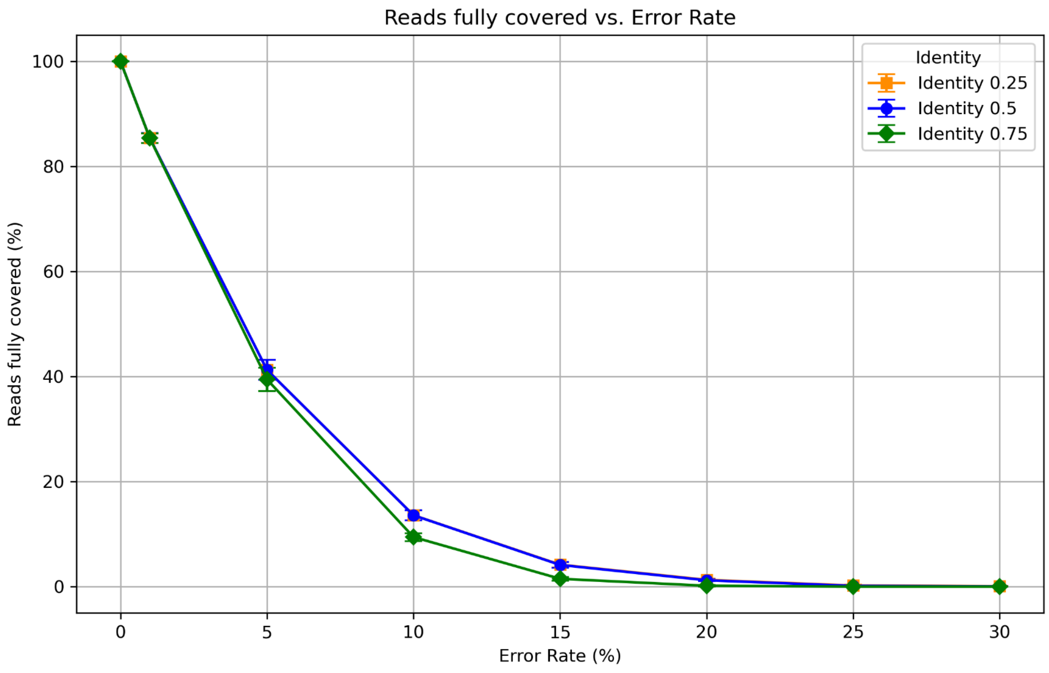

Supplementary figure 6. Fraction of reads fully covered for n = 1 with different identity values. For error rates <5 % the rate of fully covered reads decreases fast, becoming close to zero when reaching 20% error rate.

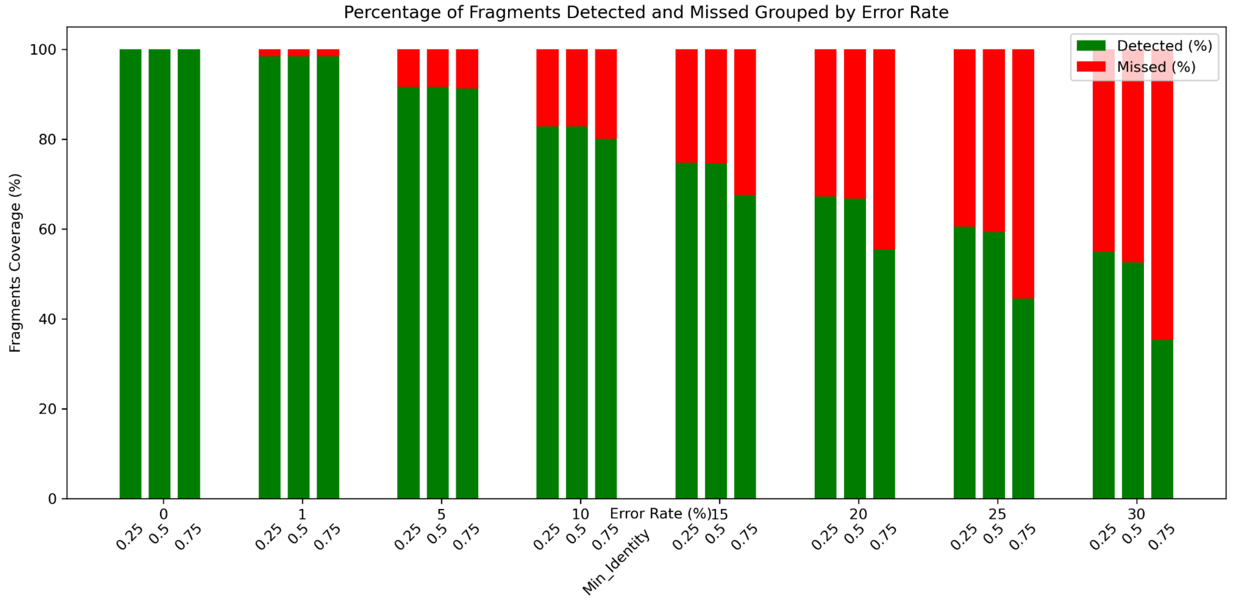

Supplementary figure 7. Fraction of fragments covered for n = 1 with different identity values.

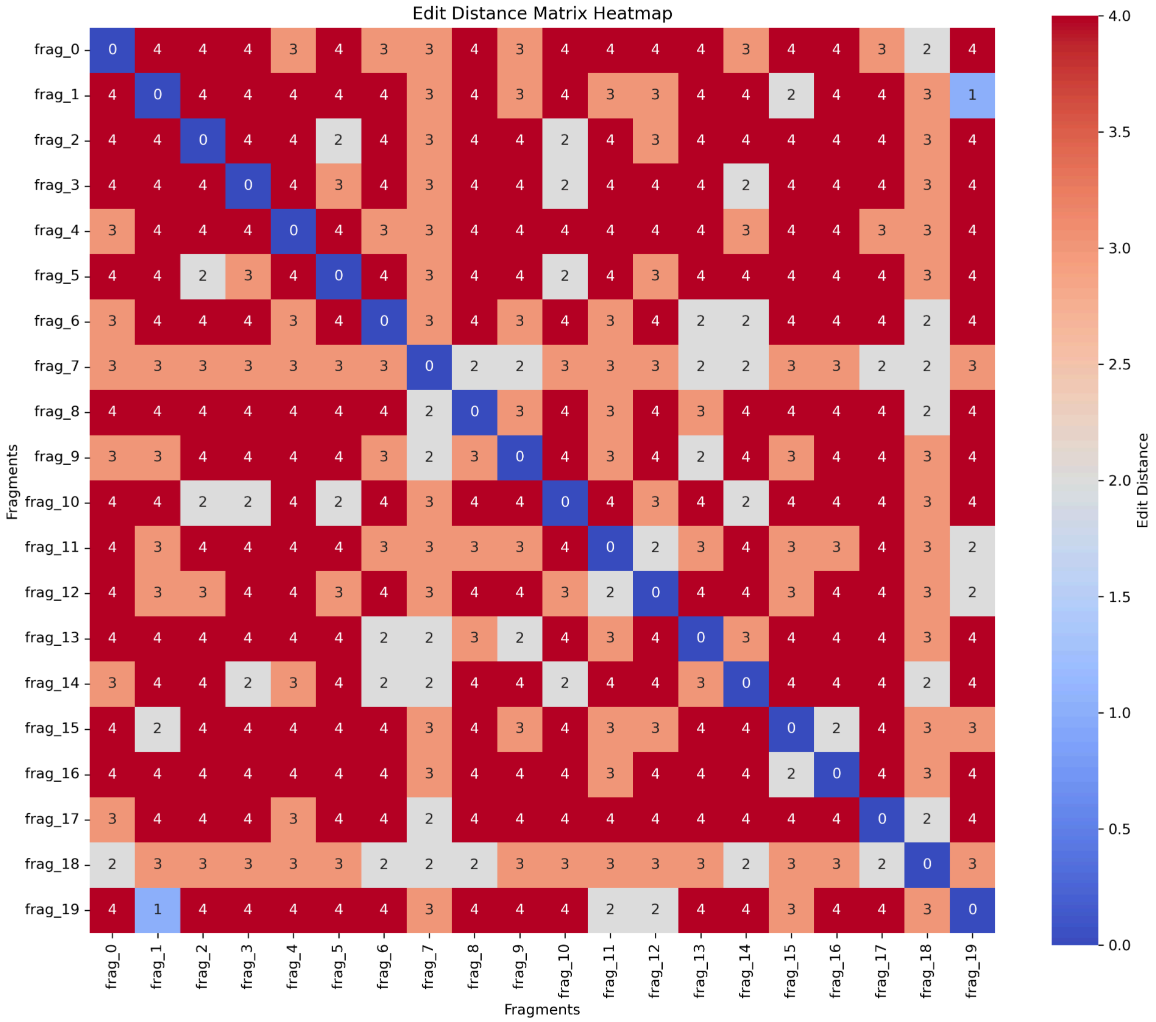

Supplementary figure 8. Edit distances of simulated fragments for n = 2.

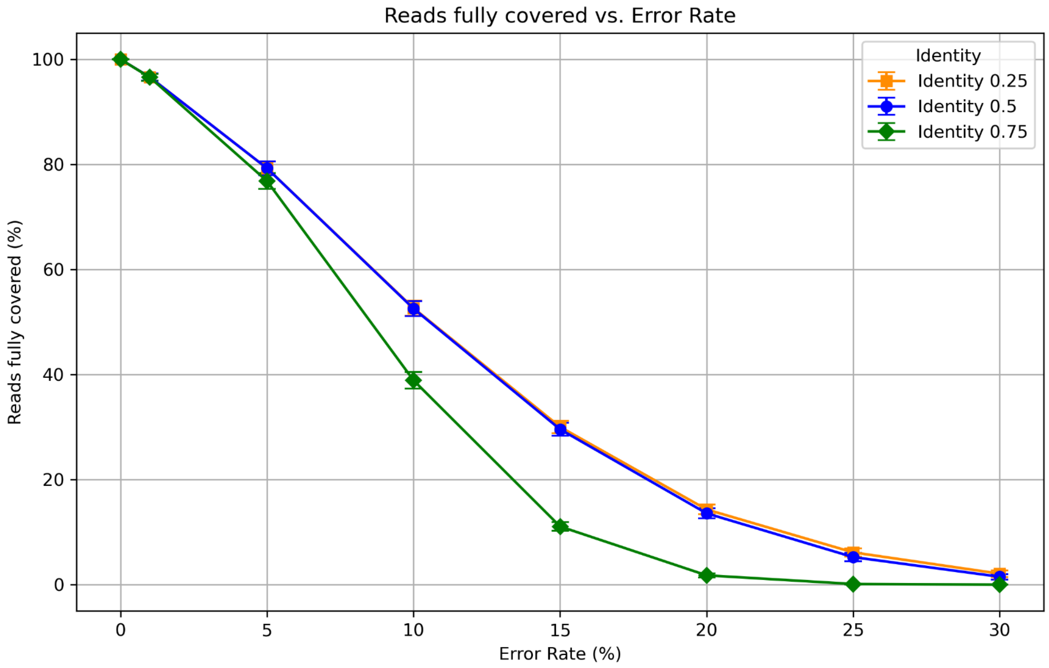

Supplementary figure 9. Percentage of reads fully covered for n = 2.

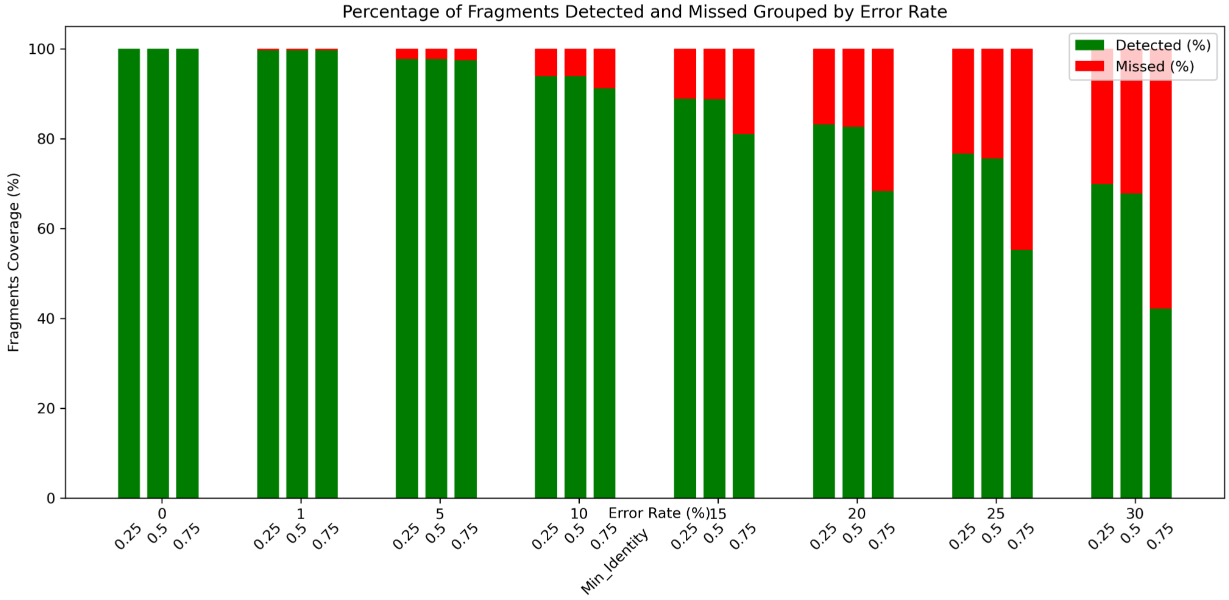

Supplementary figure 10. Percentage of fragments covered for n = 2.

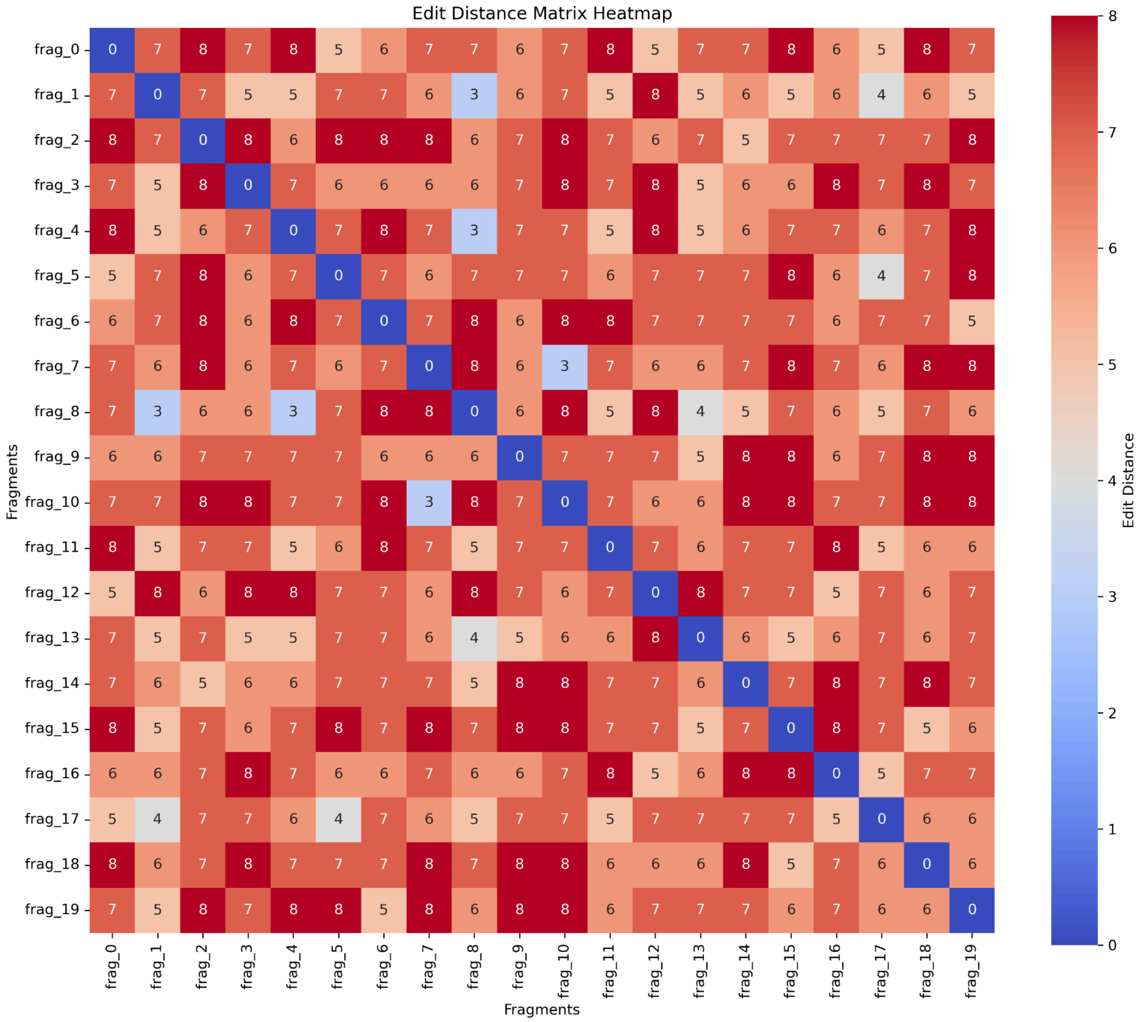

Supplementary figure 11. Edit distances of simulated fragments for n = 4.

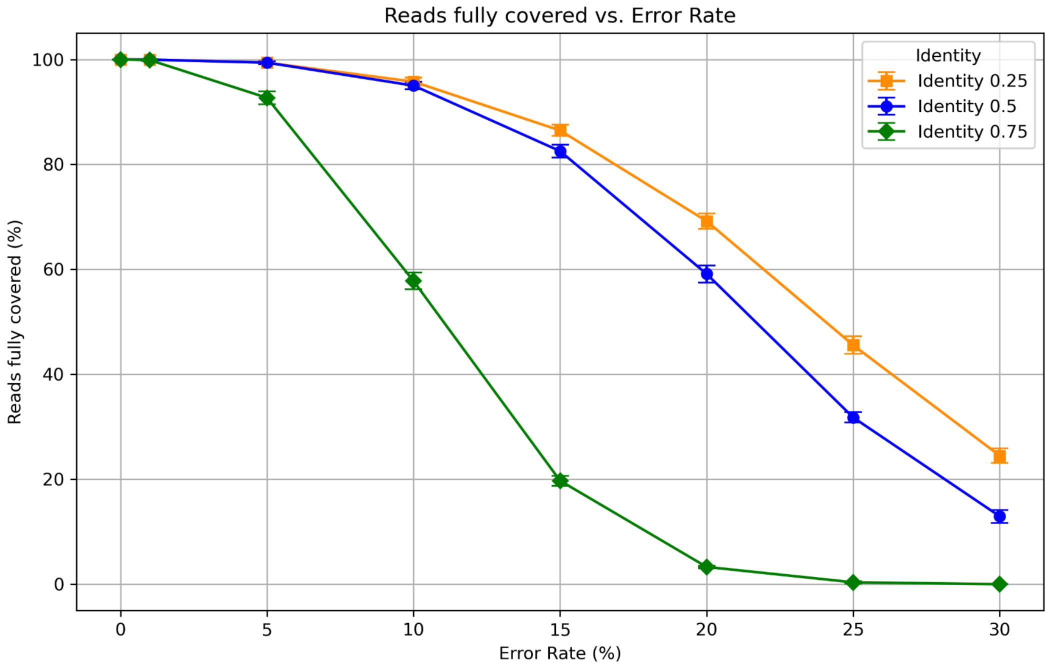

Supplementary figure 12. Percentage of reads fully covered for n=4.

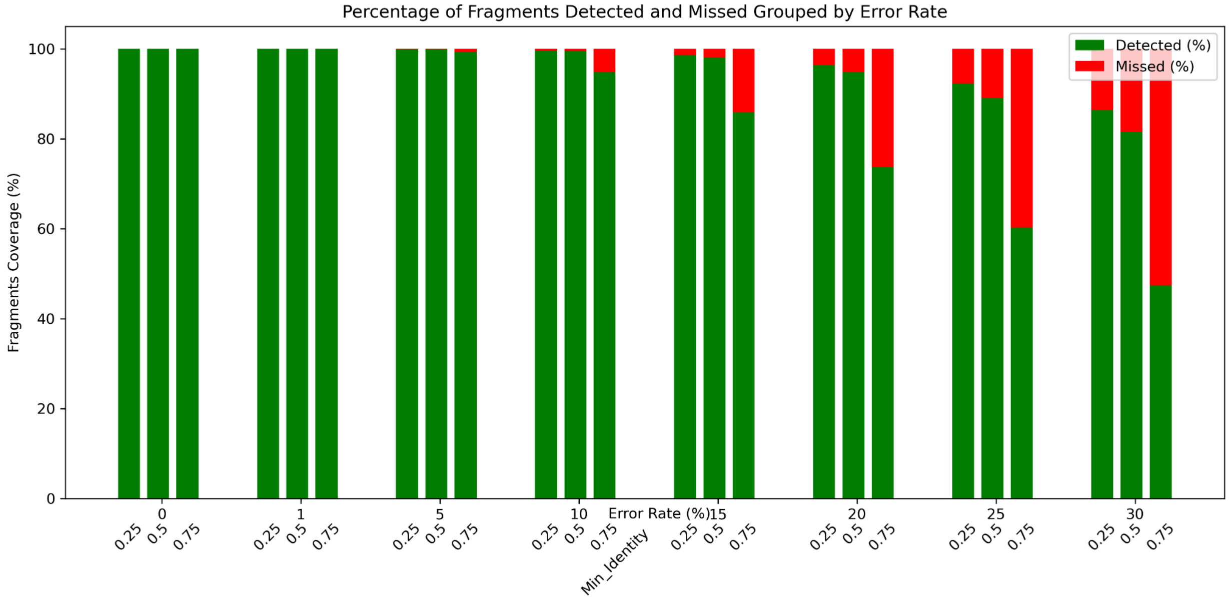

Supplementary figure 13. Percentage of fragments covered for n=4.

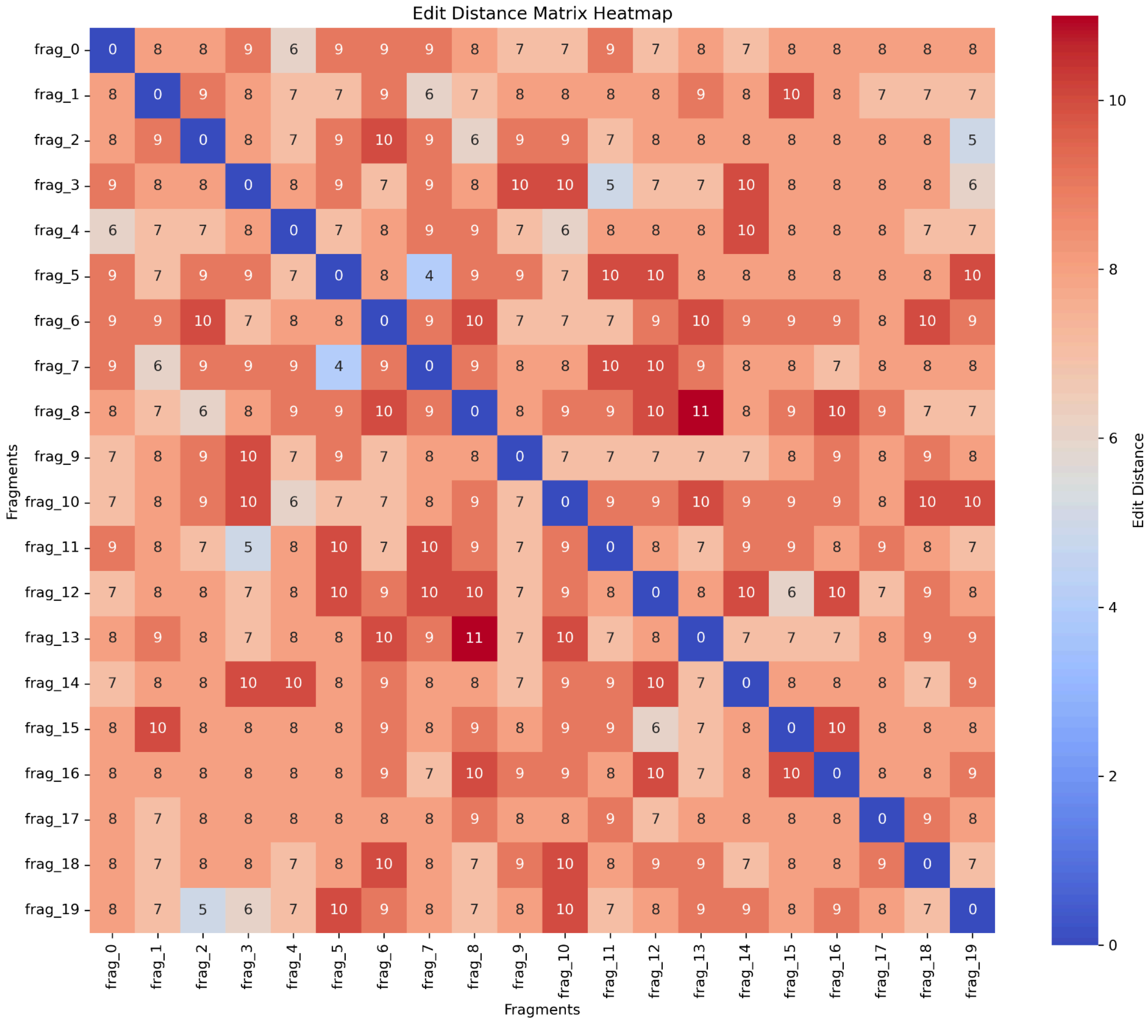

Supplementary figure 14. Edit distances of simulated fragments for n = 6. When comparing the edit distance heatmaps for each value of n to the edit distance heatmap of the fragments used for the SIM0-SIM30 experiments the closest resemblance of the edit distance patterns can be found with this heatmap.

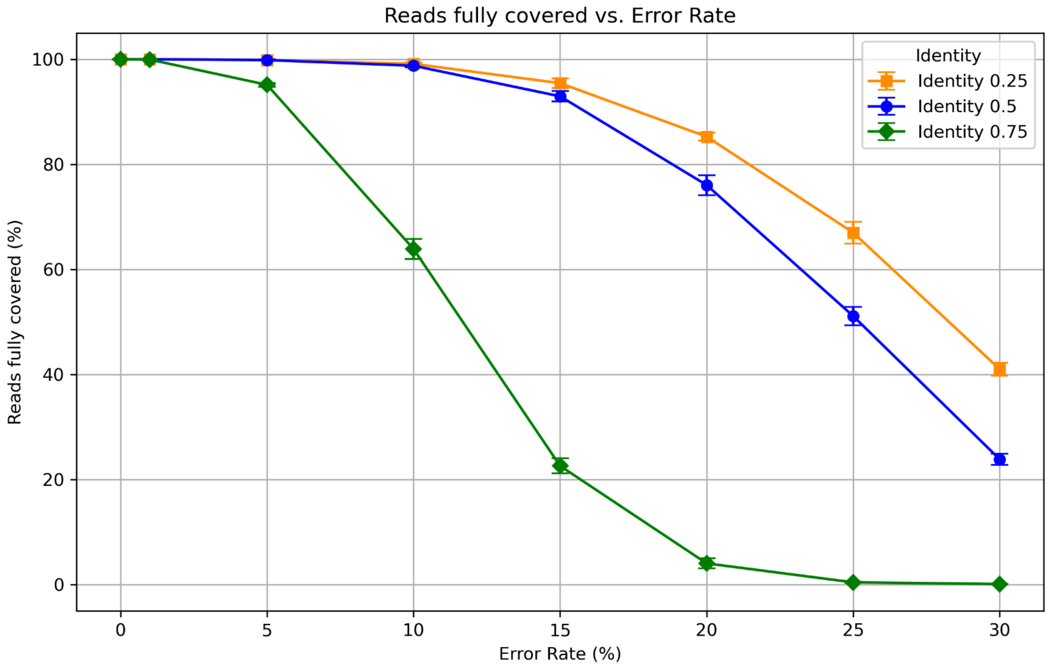

Supplementary figure 15. Percentage of reads fully covered for n = 6.

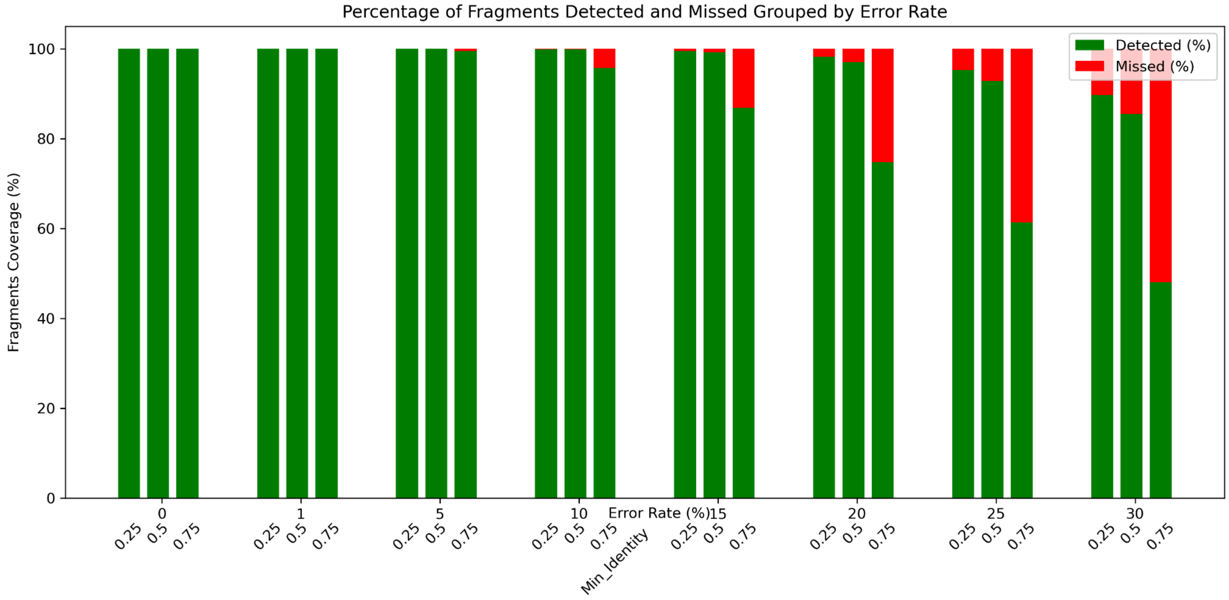

Supplementary figure 16: Percentage of fragments covered for n = 6.

Supplementary Table 1. Percentage of correct and incorrect fragment coverings out of the total time the fragment was assigned to a location using an identity setting of 0.75 for cONcat. The column "unassigned" shows the number of times a read consisted of the fragment, but it was not detected.

Fragment ID	% correct	% incorrect	unassigned
End_primer	95.7	4.3	12
End_primer_RC	100.0	0.0	19
Start_primer	98.0	2.0	9
Start_primer_RC	100.0	0.0	14
fragmentA_10	98.6	1.4	27
fragmentA_10_RC	98.0	2.0	24
fragmentA_11	99.5	0.5	17
fragmentA_11_RC	98.3	1.7	22
fragmentA_12	100.0	0.0	20
fragmentA_12_RC	99.5	0.5	13
fragmentA_13	100.0	0.0	24
fragmentA_13_RC	98.5	1.5	19
fragmentA_14	100.0	0.0	19
fragmentA_14_RC	99.5	0.5	16
fragmentA_15	100.0	0.0	21
fragmentA_15_RC	100.0	0.0	9
fragmentA_16	100.0	0.0	12
fragmentA_16_RC	100.0	0.0	11
fragmentA_17	100.0	0.0	19
fragmentA_17_RC	100.0	0.0	15
fragmentA_18	100.0	0.0	18
fragmentA_18_RC	100.0	0.0	15

Fragment ID	% correct	% incorrect	unassigned
fragmentA_19	100.0	0.0	12
fragmentA_19_RC	100.0	0.0	13
fragmentA_20	100.0	0.0	13
fragmentA_20_RC	100.0	0.0	12
fragmentB_10	100.0	0.0	16
fragmentB_10_RC	97.8	2.2	17
fragmentB_11	97.4	2.6	18
fragmentB_11_RC	98.9	1.1	27
fragmentB_12	99.5	0.5	32
fragmentB_12_RC	99.5	0.5	8
fragmentB_13	100.0	0.0	8
fragmentB_13_RC	99.4	0.6	24
fragmentB_14	99.5	0.5	16
fragmentB_14_RC	100.0	0.0	20
fragmentB_15	100.0	0.0	17
fragmentB_15_RC	100.0	0.0	17
fragmentB_16	100.0	0.0	18
fragmentB_16_RC	100.0	0.0	12
fragmentB_17	100.0	0.0	14
fragmentB_17_RC	100.0	0.0	9
fragmentB_18	100.0	0.0	12
fragmentB_18_RC	100.0	0.0	16
fragmentB_19	100.0	0.0	19
fragmentB_19_RC	100.0	0.0	16
fragmentB_20	100.0	0.0	12
fragmentB_20_RC	100.0	0.0	8

Supplementary Table 2. Percentage of correct and incorrect fragment coverings out of the total time the fragment was assigned to a location using an identity setting of 0.5 for cONcat. The column "unassigned" shows the number of times a read consisted of the fragment, but it was not detected.

Fragment ID	% correct	% incorrect	unassigned
End_primer	78.0	22.0	4
End_primer_RC	99.5	0.5	11
Start_primer	78.3	21.7	3
Start_primer_RC	100.0	0.0	5
fragmentA_10	91.8	8.2	9
fragmentA_10_RC	92.2	7.8	17
fragmentA_11	93.1	6.9	8
fragmentA_11_RC	90.9	0.1	11
fragmentA_12	95.6	4.4	7
fragmentA_12_RC	95.1	4.9	5
fragmentA_13	97.5	2.5	9
fragmentA_13_RC	98.1	1.9	9
fragmentA_14	98.4	1.6	10
fragmentA_14_RC	98.0	2.0	5
fragmentA_15	99.0	1.0	15
fragmentA_15_RC	98.4	1.6	6
fragmentA_16	100.0	0.0	5
fragmentA_16_RC	99.0	1.0	5
fragmentA_17	98.5	1.5	9
fragmentA_17_RC	100.0	0.0	9
fragmentA_18	99.0	1.0	10
fragmentA_18_RC	100.0	0.0	8

fragmentA_19	99.5	0.5	7
fragmentA_19_RC	99.5	0.5	8
fragmentA_20	100.0	0.0	5
fragmentA_20_RC	100.0	0.0	6
fragmentB_10	95.5	4.5	11
fragmentB_10_RC	90.0	10.0	6
fragmentB_11	94.6	5.4	9
fragmentB_11_RC	93.5	6.5	6
fragmentB_12	96.3	3.7	15
fragmentB_12_RC	95.6	4.4	6
fragmentB_13	96.8	3.2	3
fragmentB_13_RC	95.2	4.8	16
fragmentB_14	98.5	1.5	10
fragmentB_14_RC	97.6	2.4	10
fragmentB_15	99.0	1.0	11
fragmentB_15_RC	99.5	0.5	11
fragmentB_16	99.5	0.5	9
fragmentB_16_RC	99.0	1.0	3
fragmentB_17	99.1	0.9	7
fragmentB_17_RC	100.0	0.0	3
fragmentB_18	100.0	0.0	5
fragmentB_18_RC	99.1	0.9	6
fragmentB_19	99.5	0.5	3
fragmentB_19_RC	100.0	0.0	7
fragmentB_20	100.0	0.0	7
fragmentB_20_RC	100.0	0.0	8

Supplementary Table 3. Percentage of correct and incorrect fragment coverings out of the total time the fragment was assigned to a location using an identity setting of 0.25 for cONcat. The column “unassigned” shows the number of times a read consisted of the fragment, but it was not detected.

Fragment ID	% correct	% incorrect	unassigned
End_primer	73.7	26.3	3
End_primer_RC	99.5	0.5	11
Start_primer	74.6	25.4	1
Start_primer_RC	100.0	0.0	5
fragmentA_10	90.7	9.3	9
fragmentA_10_RC	91.8	8.2	15
fragmentA_11	93.1	6.9	8
fragmentA_11_RC	90.5	9.5	10
fragmentA_12	95.6	4.4	7
fragmentA_12_RC	94.7	5.3	5
fragmentA_13	96.5	3.5	9
fragmentA_13_RC	98.1	1.9	9
fragmentA_14	98.4	1.6	10
fragmentA_14_RC	97.5	2.5	5
fragmentA_15	99.0	1.0	15
fragmentA_15_RC	98.4	1.6	6
fragmentA_16	100.0	0.0	5
fragmentA_16_RC	99.0	1.0	5
fragmentA_17	98.5	1.5	9
fragmentA_17_RC	100.0	0.0	9
fragmentA_18	98.9	1.1	10
fragmentA_18_RC	100.0	0.0	8

Fragment ID	% correct	% incorrect	unassigned
fragmentA_19	99.5	0.5	7
fragmentA_19_RC	99.5	0.5	8
fragmentA_20	100.0	0.0	5
fragmentA_20_RC	100.0	0.0	6
fragmentB_10	95.5	4.5	10
fragmentB_10_RC	88.4	11.6	5
fragmentB_11	94.1	5.9	9
fragmentB_11_RC	93.5	6.5	6
fragmentB_12	95.9	4.1	15
fragmentB_12_RC	95.6	4.4	6
fragmentB_13	96.8	3.2	3
fragmentB_13_RC	94.2	5.8	15
fragmentB_14	98.4	1.6	9
fragmentB_14_RC	97.6	2.4	10
fragmentB_15	99.0	1.0	11
fragmentB_15_RC	99.5	0.5	11
fragmentB_16	99.5	0.5	9
fragmentB_16_RC	99.0	1.0	3
fragmentB_17	99.1	0.9	7
fragmentB_17_RC	100.0	0.0	3
fragmentB_18	100.0	0.0	5
fragmentB_18_RC	99.1	0.9	6
fragmentB_19	99.5	0.5	3
fragmentB_19_RC	100.0	0.0	7
fragmentB_20	100.0	0.0	7
fragmentB_20_RC	100.0	0.0	8
